# Enhancing safe medication use in home care: insights from informal caregivers

**DOI:** 10.3389/fmed.2024.1494771

**Published:** 2024-11-05

**Authors:** Eva Gil-Hernández, Pura Ballester, Mercedes Guilabert, Alicia Sánchez-García, Daniel García-Torres, María Pilar Astier-Peña, María Teresa Gea-Velázquez de Castro, Ángel Cobos-Vargas, Pastora Pérez-Pérez, Irene Carrillo, Ana María Fernández-Navascués, José Joaquín Mira

**Affiliations:** ^1^Atenea Research Group, Foundation for the Promotion of Health and Biomedical Research, Sant Joan d'Alacant, Spain; ^2^Pharmacology Department, San Antonio Catholic University, Murcia, Spain; ^3^Department of Health Psychology, Miguel Hernández University of Elche, Elche, Spain; ^4^Primary Care Quality Unit, Territorial Health Authority, Camp de Tarragona, Health Institut of Catalonia, Barcelona, Spain; ^5^Department of Public Health, History of Science and Gynaecology, University Miguel Hernández of Elche, Alicante, Spain; ^6^Intensive Care Unit, Patient Safety Leader, San Cecilio University Hospital, Granada, Spain; ^7^Unidad Territorial II, Provincia San Juan de Dios de España, Seville, Spain; ^8^Servicio de Efectividad y Seguridad Asistencial, Servicio Navarro de Salud - Osasunbidea, Pamplona, Spain

**Keywords:** home care, informal caregivers, medication error, patient safety, chronic disease, cross-sectional survey

## Abstract

**Objectives:**

To investigate the factors influencing medication errors made by informal caregivers while providing care at home.

**Methods:**

A cross-sectional study based on an online survey, which included both structured and open-ended questions, was conducted in Spain. The survey comprised 49 questions to collect self-reported avoidable medication errors made by caregivers at home.

**Results:**

A total of 685 caregivers participated in the survey, with 346 considered qualified (having received >20 h of training). On average, 13.5 (SD 38.2, 95% CI 10.5–16.5) errors per caregiver per year were self-reported. Errors were more prevalent among non-qualified caregivers, males, direct relatives of the care recipient, those with external occupations, or those who used external aids.

**Conclusion:**

Errors made by informal caregivers occur more frequently than expected, and recognizing these errors remains a challenge. Training is essential for creating safer care environments by increasing awareness of error sources and the risks associated with medication. Recipients’ direct relatives should receive appropriate training, considering differences between male and female caregivers. Associations and companies within the care economy sector should prioritize the creation of safer home care environments as a key objective.

## 1 Introduction

Nowadays, population aging is one of the challenges that we face, particularly concerning in countries as Japan, Italy, Greece, Germany, Korea, and Spain (1). The number of individuals aged 65 and over (approximately 1 billion worldwide), coupled with those classified under the dependency ratio, is also growing [e.g., 75.3% in Japan, 67% in Italy, and 63.1% in Germany (2)]. A third of the elderly population will require a caregiver to assist with daily activities, alongside 1.3 billion people experiencing significant disability, representing 16% of the world’s population and a high demand for care (3, 4). The prevalence of multiple chronic conditions and the need for polypharmacy (taking more than 5 medications simultaneously on a daily basis) are concentrated within this age group (5). In many countries, efforts are being made to promote seniors’ autonomy, aiming to prevent their admission to residential facilities (6–8). This aligns with the growing trend of people in developed countries who wish to age in their own homes (9). Since health and social services cannot meet all demands, informal caregivers play a crucial role (10).

In this context, the responsibilities of informal caregivers have been increasing due to escalating healthcare demands and complexity. These demands include managing diverse medication regimens daily and attending to progressively intricate care needs, requiring heightened dedication. These caregiving tasks have been predominantly undertaken by women, due to a gender gap that continues to persist (11). This new context invites to reflect on how to address the safety of patients receiving care in their homes from informal caregivers, males and females, being this an existing gap in the body of knowledge. The more severe the condition of the person receiving care (multiple diseases or medications), the higher the chances of making an error, which triples if there are multiple caregivers at home (12). This leads to growing concern among informal caregivers about the safety of those they care for, prompting them to come up with homemade solutions and learn from personal experience to avoid errors (13, 14).

Common errors include missed dosing, food interactions, confusion between medications, forgetfulness, and errors in storing or administering expired medications in both nursing homes (15), and home settings. Additionally, other risks such as infection due to inadequate practices, misuse of medical devices, choking incidents, ulcers, and bodily harm from improper mobility exist. Medication errors are a negative experience for both the care recipient and the caregiver and increase healthcare expenses (e.g., visits to the emergency room or new treatments) (16, 17). The literature has focused on the frequency, causes, and prevention of errors in healthcare facilities (18), as well as on the number of unintentional (19) mistakes made by patients with medication. However, the situation concerning informal caregivers has been less studied.

Studies on household caregiving reveal that 73.1% of adults receiving care experience a medication error annually according to STOP criteria (20), with 5% of these with errors resulting in serious consequences. Other studies described that up to 88% had a potentially inappropriate drug prescription (21, 22). Few studies to date have focused on informal caregivers. Preliminary studies indicate a wide range from 2 to 70% in the frequency with which informal caregivers report unintentional errors while caring for dependent individuals at home (14, 23, 24). These data suggest that informal caregivers are likely to encounter doubts and unintentionally make errors when providing care at home. Involving informal caregivers in safety practices during medication administration is crucial (23, 25). Despite these data, the contribution of informal caregivers to patient safety at home has been poorly studied, leaving several open questions regarding adequate care in this setting. The nature of these errors and the factors influencing safety in home environments where patients are cared for by informal caregivers (such as patient profile, dedication to the act of caregiving, training received or gender) have only recently begun to be considered. Identifying areas where patient safety is compromised at home is essential, as it can positively impact on patients’ prognosis, medication, safety, and healthcare services usage (26).

This study explores the critical role of informal caregivers in creating a safe environment at home for their care recipients, aiming to delve beyond the knowledge of medication error frequency. It aimed to examine the impact of caregiver training, full-time dedication to caregiving, utilization of devices, and the caregiver-recipient relationship on the occurrence of errors. Given that the number of women caring for family members is significantly higher (27), the study also explored whether men encounter more difficulties than women when providing care. Certain factors are expected to significantly reduce medication errors in home care settings. These include more intensive and targeted training for informal caregivers, the caregiver’s full-time commitment to the role, the use of assistive tools like pill organizers, and, due to their often-greater caregiving experience associated with traditional gender roles, women are likely to have a lower rate of medication errors. These considerations guided our analyses, allowing us to explore the challenges informal caregivers face in-depth. By addressing these key aspects, this research endeavors to shed light on the challenges faced by informal caregivers and provide new insights for effective strategies to promote patient safety and autonomy within non-institutional care settings.

## 2 Methods

### 2.1 Study design

A cross-sectional study based on an online survey was conducted in Spain. The field study was conducted between April to June 2022. This manuscript has been built following the CHERRIES checklist (28).

### 2.2 Ethical consideration

This project obtained approval from the Ethics Committee board of Sant Joan d’Alacant University Hospital in December 2021 (project CODE: 21/063) and was registered by ClinicalTrials (ref. NCT05247801). The information provided to study participants emphasized that their responses would be kept confidential, participation was voluntary, and informed consent was requested upon recruitment. Given the sensitivity of information regarding errors made in the home, all personal data was excluded from the responses, and only aggregated data was used to ensure confidentiality.

### 2.3 Definitions

An informal caregiver was defined as any person responsible for administering care and prescribed medication to the adult at home, including family members or individuals hired by the recipient or relatives.

Errors were defined as all avoidable and involuntary events related to actions/omissions of medication that may imply or not harm (29).

Training encompasses various methods and channels for information dissemination, such as written instructions, instructional videos, and observing demonstrations by healthcare professionals. We define training broadly to include any form of passive or active learning provided to informal caregivers. Notably, hours of caregiving experience alone were not considered formal training.

### 2.4 Participants

All participants were aged 18 years or older and were actively caring for a person at that moment and for at least during the previous year, providing care and medications. Participants were stratified into two groups: qualified and non-qualified caregivers based on their self-reported training. We set a threshold of 20 h of training on techniques, strategies, and resources for caregiving duties. If caregivers reported at least this amount of training, they were considered qualified. This threshold aligns with the minimum limit for training courses in health schools. Health and care qualified professionals were not included. The time taken to respond was not an exclusion criterion since the respondents’ age might influence this aspect. The participants’ wish to withhold demographic data to ensure their anonymity was respected.

### 2.5 Sample size

A required sample size of 384 caregivers in each group (a total of 768) was calculated based on the formula for estimating the proportion of occurrence of a specific event in an infinite population. Parameters were established to achieve a precision of 5% in the estimation of a proportion using a 95% two-sided normal approximation confidence interval. It was assumed, based on previous studies conducted in Spain (13), that the expected proportion is 50%. Supplementary File 1 provides more information about the calculation of the sample size performed.

### 2.6 Procedure

The survey was announced, and recruitment was encouraged through invitations sent to all subscribers of a national database of caregivers registered with companies in the care economy sector and national caregiver associations. Given that males represent a small proportion of caregivers, their representation was artificially increased to 30% by inviting 3 males for every 10 females. This adjustment was implemented to ensure the validity of the data and to enable meaningful comparisons between genders, ensuring that male caregivers were sufficiently represented in the analysis. During the two-month recruitment frame, all received three times a message with study purpose and the invitation to respond.

### 2.7 Instrument

The survey content was formulated based on the researchers’ prior experience (19, 30), a review of relevant literature, and online discussions with three experts in health psychology, pharmacy, and public health. These experts contributed to a better understanding of informal caregivers’ perspectives during group sessions, helping to identify relevant issues that needed to be explored and providing their clinical experience. The readability of the questions and the suitability of the response scales were ensured with the collaboration of informal caregivers and by incorporating insights from previous studies.

These discussions aimed to synthesize their collective expertise on patient safety, medication, and caregiving errors. To refine the survey, two members of caregivers’ associations and five additional experts conducted an online pre-reading, providing feedback on its clarity, completeness, structure, and relevance. After incorporating their insights, the survey underwent evaluation for readability, completion time, and content appropriateness before dissemination to respondents.

This online form was hosted on a plain website created to allocate surveys, owned by the research group^1^ (31), where participants’ responses were automatically entered into the study database. The platform prevented multiple responses from the same IP address, but no cookies were used. Participants did not receive any incentives for participating. We did not track data regarding survey link email opening or clicks from the central server.

It comprised fixed multiple-choice and open-ended questions designed to assess: (a) care recipients’ health status, (b) safety measures to prevent errors and description of medication errors at home, and (c) caregivers’ experiences. The survey included a total of 49 items: 11 yes/no questions, 19 questions with a Likert scale structure (never during last year, once per year, once per month, twice a month, once a week, more than twice a week), 16 multiple choice questions, and three open-ended questions.

In the first part of the survey, caregivers were asked about their knowledge regarding medication indications, dosages, or storing conditions, primarily using yes/no questions. Regarding medication errors, we examined with Likert scale statements the probability of: (a) administering an incorrect dose of different medication formulations (e.g., eye drops); (b) administering a wrong medication due to a similar appearance; (c) duplicating or missing a dose and the underlying causes; (d) not following providers’ indications regarding treatment schedule or duration; (e) giving expired medication; and (f) not following medication manipulation or conservation recommendations. Each category was displayed on a single page. Some questions were conditionally displayed according to participants’ responses and respondents were allowed to change answers before submitting the responses (see Supplementary File 2 for survey content).

### 2.8 Data management

All the information presented is derived from data self-reported by the participant caregivers. The ELIXIR Research Data Management Kit (RDMkit) has been used as a guide to assure lawfulness, fairness and transparency, limitation and minimization, accuracy, storage limitation and integrity, and confidentiality on the website where this survey is hosted. The data preservation and curation were covered by FISABIO using the free and safe data storage, operated by the institution. All data from this study will be made available upon request.

First, the quality of data was analyzed for its suitability for the intended purpose. Records of caregivers who did not respond to at least 80% of the questions, who did not adequately complete the mandatory sections, or who did not provide care or medication were excluded.

### 2.9 Data analysis

After data curation, quantitative results were reported as mean and standard deviation or median and interquartile range for medication data. The total number of unintentional errors was calculated based on the self-reported frequency by caregivers over the past year. Caregivers reported the number of errors made in the past year, which were extrapolated to an annual scale using the following conversions: 1 error per year was kept as 1 error, 1 error per month was converted to 12 annual errors, 2 errors per month to 24 annual errors, 1 error per week to 54 annual errors, and more than 1 error per week was estimated at 104 annual errors. This extrapolation allowed for the standardization of responses into an annualized metric. This calculation was also performed for each type of medication administration route (e.g., inhalers, eye drops). Comparisons were made using either Fischer’s Exact Test or Chi-Square Test, with adjustments for variable dependency and significance. Bivariate statistics were used to explore differences in the impact of training, sex, relationship to the care recipient, full-time dedication, and the use of external aids for medication management. A Poisson regression model was utilized to analyze factors associated with the frequency of self-reported avoidable medication errors over the past year. Cases with missing values were excluded from the regression analysis to ensure data integrity. To mitigate confounding bias, interactions between caregiver age and gender were considered, as these factors can influence the likelihood of errors. This approach allows to determine whether the observed differences are truly attributable to caregiver training or rather to their experience. Additionally, comparisons were conducted by segmenting the sample on the reported level of training to examine the error frequency according to variables such as caregiver sex, relationship to the care recipient, full-time dedication, and use of external aids for medication management.

Qualitative variables were presented as percentages after grouping responses into categories based on their similarity. Two researchers (EGH and PB) performed the grouping, with a third researcher (JM) participating in case of doubts regarding the correct classification of participants’ responses. Open-ended questions were analyzed considering the frequency of mentioned mistakes and calculating the percentage of responses for each type of error. Responses were condensed, and the most frequently mentioned topics were summarized.

Data statistical analysis was performed using SPSS es 28.0.0.0 and RStudio Desktop 2022.02.3. A significant difference was considered when the *p* value was <0.05.

## 3 Results

### 3.1 Participants

A total of 747 caregivers responded to the online survey. Sixty-two were excluded due to an insufficient number of questions answered. Consequently, 685 participants were included, achieving a response rate of 89.2% (685 out of 768 expected). Among the included participants, 530 were women (87.5%; Table 1). Qualified caregivers administered a mean of 8.7 different medications (95% CI 4.4–12.9) per day, whereas non-qualified caregivers administered 6.4 different medications (95% CI 6.0–6.8) per day. When comparing qualified and non-qualified caregivers’ groups, no significant differences were found in terms of sex (*p* = 0.999), age (*p* = 0.546), nationality (*p* = 0.258), co-existence with other caregivers (*p* = 0.073), full-time dedication (*p* = 0.303), or use of external aids for medication management (*p* = 0.504). However, a greater proportion of non-qualified caregivers were care recipients’ relatives compared to the qualified caregivers (*p* < 0.001), and more qualified caregivers were found to care for multiple recipients simultaneously (professionals 43.2%) and non 40.5% (*p* = 0.585). There was no significant association between recipients taking more than 10 medications per day and being primarily cared for by women (18.1% of care provided by women and 21.1% by men, *p* = 0.528).

**Table 1:** Sample sociodemographic data.

Variables	*N*	Qualified caregivers *N* (%)	*N*	Non-qual0ified caregivers *N* (%)	*N*	Total informal caregivers *N* (%)
Years (mean, SD)	340	51.1 (14.7)	258	51.6 (13.9)	598	51.4 (14.3)
Females	345	302 (87.5)	261	228 (87.4)	606	530 (87.5)
Spanish nationality	345	213 (61.7)	260	173 (66.5)	605	386 (63.8)
Undergraduate level of studies	344	125 (36.3)	255	126 (49.4)	599	251 (41.9)
Full-time caregiving	345	293 (84.9)	253	207 (81.5)	598	500 (83.5)
Not direct relative of the care recipient	343	50 (14.6)	255	68 (26.6)	598	118 (19.7)
Cares several people simultaneously	345	119 (34.5)	253	81 (32)	598	200 (33.4)
Sole caregiver for the individual	345	183 (53.0)	257	156 (60.7)	602	339 (53.3)
Medication administered						
Eye drops	346	98 (28.3)	339	89 (26.5)	685	187 (27.4)
Inhaled	346	69 (19.9)	339	78 (23.1)	685	147 (21.5)
Injection	346	112 (32.5)	339	101 (30.0)	685	213 (32.2)
Pills, capsules, tablets	346	325 (94.2)	339	315 (93.5)	685	640 (93.8)
Syrups	346	54 (15.7)	339	53 (15.7)	685	107 (15.7)

### 3.2 Medication use and related errors at home

Pills, capsules, or tablets were the most common form of medication administration, followed by injectables (Table 1). The frequency of presentations administered by both caregivers’ groups (qualified and non-qualified) was similar (*p* = 0.504).

A total of 9,236 medication errors were self-reported by the participants over the last year, representing an average of 13.5 (SD 38.2, 95% CI 10.5–16.5) errors per caregiver per year. The most frequent errors were related to dosage administration, followed by errors related to the administration schedule (Table 2). The mean of self-reported errors with injectables per informal caregiver per year was higher than with pills, inhalers, syrups, or eye drops (Table 2).

**Table 2:** Self-reported medication errors made at home by informal caregivers in the last year.

Avoidable medication error	Caregivers who have committed this error (*N*, %)	Total frequency of errors last year
Related to dosage	307 (44.8)	5,793
Medication confusion	138 (20.1)	250
Related to schedule administration	216 (31.5)	2,042
Related to storage conditions	106 (15.5)	1,151
Any type of error	396 (57.8)	9,236
Errors based on the medication presentation	Average per caregiver per year (mean, SD)	Total frequency of errors last year
Pills, capsules, tablets	2.6 (12.5)	1,796
Injection	3.2 (17.3)	2,220
Others	2.6 (15.1)	1,777

The different factors that could be associated with the frequency of medication errors by informal caregivers are shown in Table 3, along with the average frequency of errors per caregiver per year. Receiving a higher number of training hours (qualified caregivers) also tended to decrease the probability of errors by 21 times. Being aware of the number of medications the recipient takes contributed to a 65% reduction in the probability of medication errors occurring. Similarly, being a full-time caregiver reduced the probability by 40%, and being the sole caregiver for the individual by 20%. In the same way, not using external aid for medication management was associated with a 20% higher probability of making medication errors. There is also an interaction between age and training (*p* ≤ 0.001), showing that for qualified caregivers, the probability of making errors decreases by 10% as they age. In contrast, being a direct relative of the care recipient was associated with a 30% higher probability of errors. Being male or female has no direct relation to making medication errors, but the interaction between sex and being a qualified caregiver does show a decrease (*p* ≤ 0.001), reducing the probability by 30% if the caregiver is a qualified woman.

**Table 3:** Analysis of factors associated with the frequency of medication errors by informal caregivers.

Variable	Group	*N*	Average frequency of errors per caregiver per year	Total frequency of errors last year	OR	95% CI	*p* value
Qualification	Non-qualified	339	14.3	4,832	21.3	(16.82, 27.02)	<0.001
	Qualified	346	12.7	4,404			
Sex of the caregiver	Female	530	11.6	6,155	0.8	(0.64, 1.06)	0.12842
	Male	76	19.8	1,508			
Direct relatives of the care recipient	Yes	118	20.8	2,454	1.3	(1.25, 1.41)	<0.001
	No	480	10.8	5,453			
Full-time caregiving	Yes	500	11.2	5,524	0.6	(0.57, 0.64)	<0.001
	No	98	20.6	2,019			
Sole caregiver for the individual	Yes	339	11.1	3,746	0.8	(0.80, 0.89)	<0.001
	No	263	14.8	3,901			
Be sure of all medications’ quantity	Yes	621	13.1	8,118	0.35	(0.32, 0.38)	<0.001
	No	62	18	1,117			
Using an external aid for medication management	Yes	500	12.6	6,324	1.2	(1.13, 1.30)	<0.001
	No	94	14	1,317			

Table 4 presents the types and frequency of medication errors reported, categorized by caregiver qualification concerning sex, direct relative relationship, full-time caregiving status, and use of external aids for medication management.

**Table 4:** Types and frequency of medication errors by caregiver qualification regarding sex, direct relative relationship, full-time caregiving, and use of external aids for medication management.

	*Qualified caregivers*				*Non-qualified caregivers*			
Type of medication error	Male (%, *n*/*N*)	Female (%, *n*/*N*)	*p* value	OR (95% CI)	Male (%, *n*/*N*)	Female (%, *n*/*N*)	*p* value	OR (95% CI)
Dosing errors with pills	10.2, 7/41	17.1, 29/283	0.189	1.80 (0.62, 4.63)	25.8, 8/31	11.3, 24/215	0.041	2.75 (0.96, 7.31)
Confusion with pills	9.8, 4/41	2.5, 7/283	0.038	4.23 (0.87, 17.62)	7, 2/31	6.5, 15/212	1.000	0.91 (0.10, 4.23)
Dosing errors with injected medication	35.7, 5/14	20.6, 20/97	0.301	2.12 (0.50, 8.04)	33.3, 3/9	13.2, 9/68	0.142	3.21 (0.44, 18.69)
Confusion with injected medication	0, 0/14	1, 1/97	1.000	0.00 (0.00, 269.08)	0, 0/9	2.9, 2/68	1.000	0.00 (0.00, 41.89)
Dosing errors with nebulizers	12.5, 1/8	18, 11/61	1.000	0.65 (0.01, 6.01)	16.7, 1/6	15.4, 8/52	1.000	1.10 (0.02, 11.97)
Confusion with nebulizers	0, 0/8	4.9, 3/61	1.000	0.00 (0.00, 19.86)	0, 0/6	9.6, 5/52	1.000	0.00 (0.00, 10.81)
Dosing errors with eye drops	16.7, 2/12	15.1, 13/86	1.000	1.12 (0.11, 6.24)	0, 0/7	12.5, 7/56	1.000	0.00 (0.00, 6.13)
Confusion with eye drops	0, 0/12	1.2, 1/86	1.000	0.00 (0.00, 278.29)	0, 0/7	3.6, 2/56	1.000	0.00 (0.00, 44.94)
Dosing errors with syrups	0, 0/7	12.8, 6/47	1.000	0.00 (0.00, 6.25)	0, 0/3	17.6, 6/34	1.000	0.00 (0.00, 13.64)
Confusion with syrups	0, 0/7	0, 0/47	1.000	0.00 (0.00, −)	0, 0/3	2.9, 1/34	1.000	0.00 (0.00, 438.98)
Inappropriate storing conditions	14, 6/43	7.9, 24/302	0.241	1.87 (0.59, 5.12)	6.1, 2/33	9.6, 22/228	0.749	0.61 (0.07, 2.67)
Non-compliance with the administration schedule	39.5, 17/43	32.5, 98/302	0.389	1.36 (0.66, 2.74)	42.4, 14/33	32.9, 75/228	0.327	1.50 (0.66, 3.36)
	No direct relative (%, *n/N*)	Direct relative (%, *n/N*)	*p* value	OR (95% CI)	No direct relative (%, *n/N*)	Direct relative (%, *n/N*)	*p* value	OR (95% CI)
Dosing errors with pills	93.9, 275/293	94, 47/50	1.000	0.98 (0.18, 3.54)	92.5, 173/187	98.5, 67/68	0.078	0.19 (0.00, 1.26)
Confusion with pills	5.8, 10/173	10.4, 7/67	0.261	0.53 (0.17, 1.71)	4.3, 9/275	3.3, 2/47	0.666	0.76 (0.15, 7.47)
Dosing errors with injected medication	22.6, 21/93	23.5, 4/17	1.000	0.95 (0.26, 4.42)	13.3, 8/60	25, 4/16	0.265	0.47 (0.10, 2.47)
Confusion with injected medication	1.1, 1/93	0, 0/17	1.000	- (0.00, −)	3.3, 2/60	0, 0/16	1.000	- (0.05, −)
Dosing errors with nebulizers	2.3, 2/88	22.2, 2/9	0.041	0.09 (0.01, 1.35)	16.3, 7/43	7.1, 1/13	1.000	2.30 (0.25, 113.74)
Confusion with nebulizers	0, 0/88	11.1, 1/9	0.093	0.00 (0.00, 3.99)	0, 3/46	6.5, 0/14	1.000	0.00 (0.00, −)
Dosing errors with eye drops	13.6, 12/88	33.3, 3/9	0.142	0.32 (0.06, 2.24)	10.9, 5/46	14.3, 2/14	0.661	0.74 (0.10, 8.65)
Confusion with eye drops	14.3, 2/14	33.3, 3/9	0.402	0.35 (0.02, 3.95)	39.1, 18/46	39.1, 18/46	0.111	1.00 (0.40, 2.51)
Dosing errors with syrups	11.4, 5/44	10, 1/10	1.000	1.15 (0.11, 60.54)	17.9, 5/28	14.3, 1/7	1.000	1.29 (0.11, 71.81)
Confusion with syrups	0, 0/44	0, 0/10	1.000	0.00 (0.00, −)	3.5, 1/28	0, 0/7	1.000	- (0.01, −)
Inappropriate storing conditions	8.5, 25/293	10, 5/50	0.785	0.84 (0.29, 2.96)	9.1, 17/187	10.3, 7/68	0.809	0.87 (0.32, 2.61)
Non-compliance with the administration schedule	34.4, 95/293	40, 20/50	0.332	0.72 (0.37, 1.41)	29.9, 56/187	47.1, 32/68	0.017	0.48 (0.26, 0.89)
	Not full-time dedication (%, *n*/*N*)	Full-time dedication (%, *n*/*N*)	*p* value	OR (95% CI)	Not full-time dedication (%, *n*/*N*)	Full-time dedication (%, *n*/*N*)	*p* value	OR (95% CI)
Dosing errors with pills	19.6, 10/51	9.5, 26/273	0.049	2.31 (0.92, 5.41)	22.7, 10/44	11.3, 22/195	0.052	2.30 (0.89, 5.64)
Confusion with pills	7.8, 4/51	2.6, 7/273	0.077	3.22 (0.66, 13.25)	13.6, 6/44	5.7, 11/194	0.097	2.61 (0.75, 8.29)
Dosing errors with injected medication	43.8, 7/16	18.9, 18/95	0.048	3.28 (0.91, 11.51)	12.5, 1/8	16.4, 11/67	1.000	0.73 (0.01, 6.71)
Confusion with injected medication	0, 0/16	1.1, 1/95	1.000	0.00 (0.00, 230.73)	0, 0/8	2.9, 2/68	1.000	0.00 (0.00, 47.42)
Dosing errors with nebulizers	27.3, 3/11	15.5, 9/58	0.390	2.02 (0.29, 10.77)	42.9, 3/7	12.2, 6/49	0.074	5.14 (0.61, 39.91)
Confusion with nebulizers	0, 0/11	5.2, 3/58	1.000	0.00 (0.00, 13.35)	14.3, 1/7	8.2, 4/49	0.501	1.85 (0.03, 23.57)
Dosing errors with eye drops	15.8, 3/19	15.2, 12/79	1.000	1.05 (0.17, 4.55)	33.3, 2/6	9.4, 5/53	0.144	4.61 (0.34, 43.67)
Confusion with eye drops	0, 0/19	1.3, 1/78	1.000	0.00 (0.00, 159.71)	0, 0/6	3.8, 2/53	1.000	0.00 (0.00, 50.19)
Dosing errors with syrups	25, 2/8	8.7, 4/46	0.213	3.39 (0.26, 30.64)	0, 0/4	20, 6/30	1.000	0.00 (0.00, 7.71)
Confusion with syrups	0, 0/8	0, 0/46	1.000	0.00 (0.00, −)	25, 1/4	0, 0/30	0.118	- (0.19, −)
Inappropriate storing conditions	7.7, 4/52	8.9, 26/293	1.000	0.86 (0.21, 2.63)	15.2, 7/46	8.2, 17/207	0.164	2.00 (0.66, 5.51)
Non-compliance with the administration schedule	38.5, 20/52	32.4, 95/293	0.426	1.30 (0.67, 2.49)	54.3, 25/46	30.4, 63/207	0.003	2.71 (1.35, 5.51)
	Without any aid (%, *n*/*N*)	Using at least one aid (%, *n*/*N*)	*p* value	OR (95% CI)	Without any aid (%, *n*/*N*)	Using at least one aid (%, *n*/*N*)	*p* value	OR (95% CI)
Dosing errors with pills	4.7, 2/43	12.1, 34/280	0.195	0.35 (0.04, 1.47)	15.4, 6/39	12.2, 24/196	0.601	1.30 (0.40, 3.61)
Confusion with pills	2.3, 1/43	3.6, 10/280	1.000	0.64 (0.01, 4.74)	7.7, 3/39	7.1, 14/196	1.000	1.08 (0.19, 4.17)
Dosing errors with injected medication	21.4, 3/14	22.7, 22/97	1.000	0.93 (0.15, 3.97)	23.1, 3/13	14.5, 9/62	0.426	1.75 (0.26, 8.85)
Confusion with injected medication	0, 0/14	1, 1/97	1.000	0.00 (0.00, 269.08)	0, 0/13	3.2, 2/62	1.000	0.00 (0.00, 26.02)
Dosing errors with nebulizers	66.7, 2/3	15.2, 10/66	0.076	10.59 (0.51, 668.55)	11.1, 1/9	17, 8/47	1.000	0.61 (0.01, 5.84)
Confusion with nebulizers	0, 0/3	4.5, 3/66	1.000	0.00 (0.00, 70.29)	11.1, 1/9	8.5, 4/47	1.000	1.34 (0.02, 16.12)
Dosing errors with eye drops	8.3, 1/12	16.3, 14/86	0.686	0.47 (0.01, 3.75)	7.7, 1/13	13.6, 6/44	1.000	0.53 (0.01, 5.13)
Confusion with eye drops	8.3, 1/12	0, 0/86	0.122	- (0.18, −)	0, 0/13	4.5, 2/44	1.000	0.00 (0.00, 18.39)
Dosing errors with syrups	0, 0/8	13, 6/46	1.000	0.00 (0.00, 5.21)	12.5, 1/8	18.5, 5/27	1.000	0.64 (0.01, 7.30)
Confusion with syrups	0, 0/8	0, 0/46	1.000	0.00 (0.00, −)	12.5, 1/8	0, 0/27	0.229	- (0.09, −)
Inappropriate storing conditions	7.8, 4/51	8.9, 26/293	1.000	0.87 (0.21, 2.69)	9.3, 4/43	9.2, 19/207	1.000	1.01 (0.24, 3.29)
Non-compliance with the administration schedule	21.6, 11/51	34.5, 104/293	0.055	0.50 (0.22, 1.05)	30.2, 13/43	36.2, 75/207	0.488	0.76 (0.34, 1.62)

### 3.3 Caring errors at home

Both groups of caregivers predominantly faced challenges in dealing with care recipients’ swallowing problems, dysphagia, and providing an adequate texture accordingly (Table 5). Secondly, coordinating with relatives, sharing information, and agreeing on care procedures were reported as significant sources of stress and difficult emotions. Additionally, disorder or lack of specific instructions about medication and care was identified as a source of errors.

**Table 5:** Self-reported caregivers’ sources of caring mistakes and worries.

Factor contributing to caring mistakes	Qualified caregivers (*n*, %)	Non-qualified caregivers (*n*, %)	*p* value
Food textures. Recipient has difficulties in swallowing	39, 11%	12, 3%	<0.001
No access to medical instructions	12, 3%	5, 1%	0.049
Caregiving mistakes stemming from lack of skill	14, 4%	3, 1%	0.002
Recipient’s refusal and defiant behavior toward receiving care	1, 0%	6, 2%	0.131

## 4 Discussion

### 4.1 Major findings

This study adds to the existing knowledge by examining caregiver factors that influence medication and caregiving errors in home settings. Factors such as training, sex, use of external aids, being a relative of the recipient, or dedicating full-time caregiving were considered. This is the first European study analyzing patient safety information from informal caregivers. The study involved middle-aged women caregivers attending to recipients of polypharmacy, mirroring the typical profile of informal caregivers in Spain, where 84–89% are mostly women, average age of 53, with 44% being housewives and 60% having a basic education level (32).

The results indicate that more training positively impacts medication safety. Medication errors increased with less than 20 h of training, when caregivers were relatives, especially males, and when no external aids for medication management were used. Informal caregivers with other occupations committed more medication errors, while full-time dedication to care led to fewer errors. Sharing caregiving responsibilities across multiple households also posed a higher risk of errors. Since the sample in this study mainly consisted of individuals whose primary dedication is caring for others at home, it is possible that the caregiving experience also contributed to safer medication use.

### 4.2 Relation to previous studies

These data confirm that the use of medications at home is not free from unintentional errors that can have consequences for patients (33). This study shows that medication errors rise with more complex treatment regimens (15). Injected medication errors were more frequent among non-qualified caregivers, who also had higher confusion rates with pill medications due to a lack of strategies to avoid confusion. As it has been described elsewhere, similarities in naming or spelling (sound-alike), and/or physical appearance or packaging (look-alike) are sources of confusion (34). Increasing awareness is everybody’s responsibility to decrease potential harms derived from these errors has been confirmed.

The findings align with those from other Western countries, where family members provide the majority of care. However, this study emphasizes that poor caregiver training is strongly linked to higher medication error rates (35). Our results are in line with previous publications where caregivers with limited language proficiency or health literacy tend to make more dosing errors (up to 83.1%), with the greatest odds in the group of non-qualified caregivers (36). Unqualified male caregivers made more errors in medication administration compared to females, contrasting with studies on nurses, where medication error rates were higher in the female group (37). Non-qualified male caregivers made more errors, including dosage and schedule adherence issues (38). Few authors have addressed these problems using a gender perspective. In studies on children’s informal caregivers, being male was associated with appropriate medication preparation (39). However, in light of these data, studies reporting medication errors should incorporate this novel analytic approach, where the caregiver’s gender is considered (30), or even specifically reported (40). This seems relevant if we consider that in Europe, data suggest that the life expectancy gap between men and women has narrowed, leading to a growing number of men taking on the caregiver role for their partners (41). This trend should be considered alongside studies indicating that many men feel a sense of diminished masculinity when assuming caregiving roles (42, 43). This perception, combined with a general lack of caregiving experience over the years, may result in less attention to available informational resources, potentially impacting the quality of care provided and influencing these outcomes. Further research could focus on these aspects to elucidate the reasons behind these differences, which may pose a greater risk for female care recipients than for male ones.

The literature reports that informal caregivers (for both the elderly and infants) make avoidable errors such as misdosing injected medicine and administering medication out of schedule (44, 45). This study confirms these initial findings and provides new data. Being relatives of the recipients, qualified caregivers showed increased nebulizer dosing errors, eye drop dose errors, and decreased compliance with treatment duration or storing conditions. Qualified caregivers with another occupation tended to commit more dosing errors with injected medication, confusion with oral medication, and duplicate administration. Being a full-time informal caregiver reduced errors with syrups, scheduling, and medication manipulation. Previous descriptions indicate that a person’s working status negatively impacts their error rates, especially missed doses (46).

There are strategies to reduce medication errors, such as using dispensing devices or labeling the dosage on the package (47). In this study, external aids (e.g., alarms, mobile apps, or medicine organizers) helped reduce errors with inhalers but did not always prevent confusion or dosage errors. Those cautious with medication use and aware of potential risks are more likely to use these aids, acknowledging that errors can still occur. This is consistent with previous findings (30). While mobile apps generally meet caregivers’ needs, increasing awareness and providing external strategies to reduce caregiving issues is crucial.

Regarding caregiving error causes and challenges, caregivers experienced problems with food textures, recipients with swallowing problems and dysphagia, and poor communication among caregivers and family members (36). Regardless of their training, both groups reported these issues. To reduce care errors in chronic patients and assess individual needs, some tools have been developed in Spain (48). Researchers have raised concerns about caregivers’ limited knowledge of dysphagia and related care actions (49), which is still an active research field for medication errors in hospital settings, where providers are struggling with dose (50) and formula (51) adaptation to provide a suitable treatment in patients with dysphagia. Enhancing effective communication among everyone involved in recipients’ care is essential, as many errors stem from unfamiliarity with medication or care processes (52).

### 4.3 Implications for practice

This study provides insights for designing interventions to prevent avoidable mistakes at home and create safer environments. For example, implement tailored training for informal caregivers, especially focused on medication administration (oral pills and injections), recognizing common errors, and understanding medication schedules. Managing food textures and dysphagia or avoid falling has proven to be a common challenge, and access to visual information on how to address these issues could be one of the priorities.

The training approach for informal caregivers should be specifically tailored to address the gaps among male elderly caregivers and those with external occupations who dedicated a limited time for caregiving, as the study highlights their increased likelihood of committing errors.

Create public resources addressing the emotional impact of caregiving and teaching caregivers coping mechanisms to target and reduce stress and design gender-sensitive support programs recognizing the specific challenges faced by male caregivers. Policy makers, social workers and clinicians should consider the factors analyzed in this study when designing more personalized information and instructions that informal caregivers need to carry out their tasks at home. This need for personalized training aligns with what has been stated in recent publications, where the increasing decline in the caregiver-to-patient ratio is already supported by artificial intelligence, helping to detect hazards or abnormal patterns in the living ambient (53).

The findings can be extended to other European countries, contributing to a detailed map of home care needs and helping policymakers develop resources and support materials to enhance safety at home.

### 4.4 Limitations

The study relied on self-reported data, which may include biases and inaccuracies. Since we relied on participants’ recollections, their responses may not fully reflect reality and may also be subject to social desirability bias. The study is not free from the risk of declarative bias, as some respondents might be more honest than others (e.g., struggling to admit their errors). Furthermore, past caregivers’ errors were self-reported, so the extent to which these self-reported errors correlated with actual errors is unknown. The recall of error occurrences may differ between recent episodes and those further in the past. Additionally, caregiver-to-caregiver communication has been pointed out as a contributory factor to errors (23). When interpreting the results, it is important to consider that the group of non-qualified caregivers had higher rates of care recipients’ relatives than the qualified group. Also, since men are underrepresented among informal caregivers, we oversampled male participants to facilitate gender comparisons. However, this may limit the study’s ability to generalize the findings, but will contribute to expanding knowledge in the remaining gaps of the field.

The sample size was not large enough for high statistical power, and there was a bias in the information shared by multiple caregivers. The study’s cross-sectional nature limits the analysis of cause-and-effect relationships. Social desirability bias and declarative bias may have affected the accuracy of reported errors. The study also did not account for whether support devices were used correctly. The methodology used does not allow for causal relationships to be established, as it is lacking a longitudinal follow-up.

### 4.5 Suggestions for further research

Future studies should explore the frequency of medication mistakes at home beyond self-reporting limitations. Analyzing the impact of training content, intensity, and duration on caregiving safety is necessary. Research should also address the emotional impact of avoidable errors on caregivers, particularly family members, and the direct or indirect costs of these errors on the healthcare system. Future efforts should aim to gain a more realistic view of caregivers’ situations, as there may be selection bias in this study, especially when caregivers share their duties with others.

Further research could investigate if mistakes at home have a similar impact on informal caregivers as adverse events in professional healthcare settings. Collaborating with caregiver and patient associations and employing citizen science approaches could overcome barriers in future studies.

## 5 Conclusion

This study confirms that a greater number of avoidable errors occur at home than expected. Although most of these errors do not result in severe consequences, some can lead to modifications in the recipient’s treatment and negatively impact the caregiver’s emotional well-being, thereby compromising their ability to provide safe care. The findings suggest that certain groups—such as male caregivers, direct family members, those with limited training, and individuals juggling caregiving with external occupations—are at a higher risk of committing medication errors, particularly with pills (solid oral dosage forms) and injections, which showed the highest error rates. Additionally, caregivers require further support to safely manage issues such as food textures and dysphagia at home. There is a clear need to prioritize enhanced communication among all individuals involved in the care process to prevent misunderstandings and improve coordination. This study underscores the practical implications for healthcare policy, emphasizing the importance of developing stronger support systems for informal caregivers. These may include tailored training programs, improved access to assistive devices, and emotional and psychological support mechanisms. Future research should explore longitudinal studies combined with intervention-based approaches to evaluate the long-term effectiveness of caregiver training programs and the impact of ongoing support on reducing medication errors and improving caregiver well-being. Such efforts will provide valuable insights for designing more robust and effective strategies to support informal caregivers and enhance patient safety in non-institutional care settings.

## Data Availability

The raw data supporting the conclusions of this article will be made available by the authors, without undue reservation.

## Footnotes

^1^ https://calite.umh.es/delphis/es/
