# Supplementary material for: Enhancing safe medication use in home care: insights from informal caregivers
Source: Front Med (Lausanne). 2024 Nov 5;11:1494771. doi: 10.3389/fmed.2024.1494771 (PMC11574791; doi:10.3389/fmed.2024.1494771)
Supplement: Supplementary file 1 [file Data_Sheet_1.DOCX]

**Supplementary File 1: Sample size calculation**

From the formula of sample size calculation for estimating the proportion of occurrence of a specific event in infinite population:

$$n=\left( \frac{Z_{\alpha/2}}{\delta} \right)^{2}p(1-p)$$

Where:

**·** $\boldsymbol{n}$ = sample size

**·** $\boldsymbol{Z}_{\boldsymbol{\alpha}/\boldsymbol{2}}$ = critical value of the confidence level (95%, $Z_{\alpha/2}$ = 1.96)

**·** $\boldsymbol{p}$ = expected proportion (0.5)

**·** $\boldsymbol{\delta}$ = estimation precision (5%)

$\boldsymbol{n}=\left( \frac{1.96}{0.05} \right)^{2}0.5\left( 1-0.5 \right)=384.16\cong\boldsymbol{384 participants in each population}$
