# Supplementary material for: Enhancing safe medication use in home care: insights from informal caregivers
Source: Front Med (Lausanne). 2024 Nov 5;11:1494771. doi: 10.3389/fmed.2024.1494771 (PMC11574791; doi:10.3389/fmed.2024.1494771)
Supplement: Supplementary file 2 [file Data_Sheet_2.DOCX]

**Supplementary File 2: Questionnaire for informal caregivers**

**INFORMATION ABOUT MEDICATION**

1. How many different medicines does the person you care for take per day?

*Answer:*

1. Do you know what all the medicines the person you care for takes are for?

- Yes
- No

1. ¿Are you sure you know how much of all the medicines the person you are caring for needs to take each day?

- Yes
- No

1. ¿ Does the person you are caring for take a needle medicine (e.g. insulin for diabetes)?

- Yes
- No *[Skip to No. 6]*

1. How many injected medications does the person you are caring for take?

*Answer:*

1. ¿ Does the person you are caring for use any nebulisers (e.g. for asthma)?

- Yes
- No *[Skip to No. 8]*

1. How many inhaled medications does the person you are caring for take?

*Answer:*

1. Does the person you are caring for take any pills/capsules/tablets?

- Yes
- No *[Skip to No. 10]*

1. How many pills, capsules, tablets does the person you are caring for take?

*Answer:*

1. Does the person you are caring for take any syrups?

- Yes
- No *[Skip to No. 12]*

1. How many syrups does the person you are caring for take?

*Answer:*

1. Does the person you are caring for take any eye drops (e.g. for glaucoma)?

- Yes
- No *[Skip to No. 14]*

1. How many eye drops does the person you are caring for take?

*Answer:*

**INFORMATION ABOUT MEDICATION FAILURES AT HOME**

1. In the past year, have you injected the person you care for more or less doses of the prescribed medication?
   - N/A
   - Never happened to me
   - It has happened to me once in the past year
   - It has happened to me once a month
   - It has happened to me twice a month
   - Has happened to me once a week
   - It has happened to me more than twice a week, during all days of treatment
2. Are you aware that you have given the person you care for the wrong subcutaneous injection because it looks very similar to another one that you also take or that is in the medicine cabinet at home?
   - N/A
   - Never happened to me
   - It has happened to me once in the past year
   - It has happened to me once a month
   - It has happened to me twice a month
   - Has happened to me once a week
   - It has happened to me more than twice a week, during all days of treatment
3. In the past year, have you given the person you care for more or less doses of medication to inhale?
   - N/A
   - Never happened to me
   - It has happened to me once in the past year
   - It has happened to me once a month
   - It has happened to me twice a month
   - Has happened to me once a week
   - It has happened to me more than twice a week, during all days of treatment
4. Are you aware that you have given the caregiver the wrong nebuliser because it looks too much like another nebuliser that you also take or that is in the medicine cabinet at home?
   - Never happened to me
   - It has happened to me once in the past year
   - It has happened to me once a month
   - It has happened to me twice a month
   - Has happened to me once a week
   - It has happened to me more than twice a week, during all days of treatment
5. In the past year, have you given the person you care for more or less doses of pills/ tablets/coated tablets than the prescribed medication?
   - Never happened to me
   - It has happened to me once in the past year
   - It has happened to me once a month
   - It has happened to me twice a month
   - Has happened to me once a week
   - It has happened to me more than twice a week, during all days of treatment
6. Are you aware that you have given the person you care for the wrong pill/tablet/coated tablet because it looks very similar to another pill/tablet/coated tablet that you also take or that is in the medicine cabinet at home?
   - Never happened to me
   - It has happened to me once in the past year
   - It has happened to me once a month
   - It has happened to me twice a month
   - Has happened to me once a week
   - It has happened to me more than twice a week, during all days of treatment
7. In the past year, have you given more or less doses of any syrup to the person you care for than the prescribed medication?
   - Never happened to me
   - It has happened to me once in the past year
   - It has happened to me once a month
   - It has happened to me twice a month
   - Has happened to me once a week
   - It has happened to me more than twice a week, during all days of treatment
8. Are you aware that you have given the person you care for the wrong syrup because it looks very similar to another syrup that you also take or that is in the medicine cabinet at home?
   - Never happened to me
   - It has happened to me once in the past year
   - It has happened to me once a month
   - It has happened to me twice a month
   - Has happened to me once a week
   - It has happened to me more than twice a week, during all days of treatment
9. In the past year, have you given more or less doses of any eye drops to the person you care for than the prescribed medication?
   - Never happened to me
   - It has happened to me once in the past year
   - It has happened to me once a month
   - It has happened to me twice a month
   - Has happened to me once a week
   - It has happened to me more than twice a week, during all days of treatment
10. Are you aware that you have given the person you are caring for the wrong eye drops because they look very similar to another eye drop that you also take or that is in the medicine cabinet at home?
    - Never happened to me
    - It has happened to me once in the past year
    - It has happened to me once a month
    - It has happened to me twice a month
    - Has happened to me once a week
    - It has happened to me more than twice a week, during all days of treatment
11. Have you given the caregiver the medicine twice because you could not remember if you had already given it to him/her?
    - Never happened to me
    - It has happened to me once in the past year
    - It has happened to me once a month
    - It has happened to me twice a month
    - Has happened to me once a week
    - It has happened to me more than twice a week, during all days of treatment
12. Have you forgotten to give a medication to the person you care for?
    - Never happened to me
    - It has happened to me once in the past year
    - It has happened to me once a month
    - It has happened to me twice a month
    - Has happened to me once a week
    - It has happened to me more than twice a week, during all days of treatment
13. Have you given the person in your care medication out of schedule?
    - Never happened to me
    - It has happened to me once in the past year
    - It has happened to me once a month
    - It has happened to me twice a month
    - Has happened to me once a week
    - It has happened to me more than twice a week, during all days of treatment
14. Has the treatment of the person you are caring for ended earlier or later than the doctor had recommended?
    - Never happened to me
    - It has happened to me once in the past year
    - It has happened to me once a month
    - It has happened to me twice a month
    - Has happened to me once a week
    - It has happened to me more than twice a week, during all days of treatment
15. Have you ever broken or manipulated a medicine (e.g. split a tablet) and then found out that this was not allow?
    - Never happened to me
    - It has happened to me once in the past year
    - It has happened to me once a month
    - It has happened to me twice a month
    - Has happened to me once a week
    - It has happened to me more than twice a week, during all days of treatment
16. Have you given the medication to the person you are caring for after forgetting to put it in the refrigerator and therefore at room temperature?

- It has happened to me once in the past year
  - It has happened to me once a month
  - It has happened to me twice a month
  - Has happened to me once a week
  - It has happened to me more than twice a week, during all days of treatment

1. Have you given expired medication to the person you are caring for?
   - Never happened to me
   - It has happened to me once in the past year
   - It has happened to me once a month
   - It has happened to me twice a month
   - Has happened to me once a week
   - It has happened to me more than twice a week, during all days of treatment
2. Have you given any medication to the person you care for after the time specified on the package as "Once opened, use within X days/months (e.g. one eye drop) has elapsed?
   - Never happened to me
   - It has happened to me once in the past year
   - It has happened to me once a month
   - It has happened to me twice a month
   - Has happened to me once a week
   - It has happened to me more than twice a week, during all days of treatment
3. Have you given any medication to the person you care for after not respecting the storage conditions (medication in high humidity, direct sunlight, high temperature)?
   - Never happened to me
   - It has happened to me once in the past year
   - It has happened to me once a month
   - It has happened to me twice a month
   - Has happened to me once a week
   - It has happened to me more than twice a week, during all days of treatment

**INFORMATION ON WHO PROVIDES CARE AT HOME**

1. Age.

*Answer*:

1. Sex.

- Male
- Female
- Other

1. Spanish nationality?
   - Yes
   - No
2. In which Autonomous Community do you work as a caregiver?

- Andalucía
- Aragón
- Principado de Asturias
- Baleares
- Canarias
- Cantabria
- Castilla-La Mancha
- Castilla y León
- Cataluña
- Comunidad Valenciana
- Extremadura
- Galicia
- La Rioja
- Comunidad de Madrid
- Región de Murcia
- Comunidad Foral de Navarra
- País Vasco
- Ceuta
- Melilla

1. Level of education completed.

- School graduate (16 years old)
- Basic vocational training
- Intermediate vocational training
- Higher vocational training
- Baccalaureate
- University degree
- Other:

1. Are you the sole caregiver of the adult?

- Yes
- No *[Skip to No. 40]*

1. If there are multiple carers, please specify how many carers there are in total:

*Answer:*

1. Are you a direct relative of the person you are caring for (partner, children, etc.)?

- Yes
- No

1. Is caregiving your main job?

- Yes
- No

1. Do you care for several people at the same time?

- Yes
- No *[Skip to No. 45]*

1. Please specify how many persons you care for at a time:

*Answer:*

1. Do the people you care for live together?

- Yes
- No

1. Do you use anything to help you with the administration of your medication (e.g. pill dispenser, mobile phone alarm)?

- Yes
- No

1. Has the person you care for ever refused to take medication for any of the following reasons:

- Swallowing problems
- Intolerance problems (e.g. taste)
- Acceptance problems
- Other

1. When the person in your care refuses to take medication, how do you deal with this?

- I do not give it to him/her
- I try to convince him/her by explaining the need for it
- I talk to a family member to reinforce the taking of the medication.
- I make an appointment with his family doctor to discuss the matter with him.
- Other

1. We would now like you to indicate any other issues that you think may reflect medication problems in the home, and to collect suggestions:

*Answer:*

1. Before becoming a caregiver, did you receive any specific training on health and medication issues, in addition to the training you acquired as a caregiver?:

- No
- Yes, please indicate which one:
